# *TAS2R38* gene methylation is associated with syndrome Coronavirus 2 (SARS-CoV-2) infection and clinical symptoms

**DOI:** 10.1038/s41598-025-95879-x

**Published:** 2025-04-25

**Authors:** Melania Melis, Eleonora Loi, Giacomo Aru, Giorgia Sollai, Mariano Mastinu, Lala Chaimae Naciri, Giacomo De Riu, Luigi Angelo Vaira, Giulia Costanzo, Davide Firinu, Paola Cabras, Aldo Caddori, Roberto Crnjar, Patrizia Zavattari, Iole Tomassini Barbarossa

**Affiliations:** 1 Department of Biomedical Sciences, Unit of Physiology, University of Cagliari, Monserrato, 09042 CA Italy; 2 Department of Biomedical Sciences, Unit of Biology and Genetics, University of Cagliari, Monserrato, 09042 CA Italy; 3 Department of Otorhinolaryngology, Smell & Taste Clinic, Technical University of Dresden, Dresden, 01307 Germany; 4 Maxillofacial Surgery Unit, Department of Medicine, Surgery and Pharmacy, University of Sassari, Sassari, 07100 Italy; 5 Department of Medical Sciences and Public Health, University of Cagliari, Monserrato, 09042 CA Italy; 6 Department of Internal Medicine, Hospital SS. Trinità, ATS, Cagliari, 09121 Italy

**Keywords:** TAS2R38 receptor, SNPs of *TAS2R38* gene, *TAS2R38* gene methylation, COVID-19, Methylation analysis, SARS-CoV-2

## Abstract

TAS2R38 is the T2R receptor primarily associated with the innate immune response of the respiratory system. It activates a response mediated by nitric oxide (NO), which has been shown to inhibit the replication of SARS-CoV-2. *TAS2R38* polymorphisms (SNPs) that decrease receptor functionality contributing to individual differences in susceptibility to airway infections. DNA methylation (DNAm) may affect gene expression influencing disease development, including COVID-19. We analyzed the effect of SARS-CoV-2 on the methylation pattern of *TAS2R38* (at cg25481253, a CpG site located in the coding region) during infection and after the cessation of the exposure to the virus, also considering the disease severity and *TAS2R38* SNPs. Our results showed a positive relationship between *TAS2R38* DNAm levels and disease severity in the COVID-19 patients and a return to a normal state after the infection. In addition, our results showed an association between DNAm level and the TAS2R38 genotype in participants who recovered from the disease. PAV/PAV genotypes showed lower *TAS2R38* DNAm levels than heterozygous and AVI homozygous. In conclusion, our results clearly indicate the involvement of *TAS2R38* DNAm alteration in COVID-19 severity and suggest a role of the methylation changes at cg25481253 in the regulation of the *TAS2R38* expression.

**Supplementary Information:**

The online version contains supplementary material available at 10.1038/s41598-025-95879-x.

## Introduction

Taste receptors are expressed not only in the oral cavity but also in numerous extraoral tissues, including the brain, kidneys, testes, pancreas, liver, airways and gastrointestinal tract, where they use a common chemical language^1–7^. In the oral cavity, taste receptors are the initial component responsible for perceiving and discriminating taste stimuli, which are involved in assessing the nature and quality of food^8,9^. At the same time, taste receptors of extraoral tissues are involved in a variety of non‒tasting physiological processes, that can be affected by modifications in the sensitivity or expression of these receptors^10^.

Specifically, type 2 taste receptors (T2Rs), belonging to the G-protein coupled receptors superfamily (GPCRs), can detect a variety of bitter substances^11^ and are expressed in the type II cells of taste buds in the oral cavity, where they traditionally act as a primary alert system to prevent the consumption of possible toxins^12^. Besides, a growing number of data suggest that T2Rs are extensively distributed throughout the body mediating various non-tasting functions, and that various human diseases are associated with their genetic variants^6^. Some authors investigated the potential role of T2R to act as a therapeutic target for SARS-CoV-2 symptoms and the use of bitter agonists to restore their function^13^.

The most widely studied T2R is TAS2R38. It mediates the bitter taste of the 6-n-propylthiouracil (PROP) thiourea, which is described as a stimulus marker for recognizing individual differences in taste sensitivity^14^. In addition, the diverse ability of people to taste PROP is associated with the allelic diversity of the *TAS2R38* gene, defined by three single-nucleotide polymorphisms (SNPs), which give rise to two common haplotypes. The functional variant contains the amino acids proline, alanine, and valine (PAV) and the nonfunctional variant contains alanine, valine, and isoleucine (AVI) at these same amino acid positions in the receptor protein (49, 262, 296)^15^. Likely, the loss of valine at the 296 position in the AVI form blocks the activation of the receptor by agonist binding^15,16^. It was assumed that PROP non-taster individuals are homozygous for the AVI haplotype, PROP super-tasters are homozygous for the PAV haplotype and PROP medium tasters are heterozygous^17^. *TAS2R38* SNPs also control TAS2R38‒mediated pathophysiology^6^, including susceptibility, course, and outcomes of upper respiratory infection^18,19^, chronic rhinosinusitis^20–25^, development of colorectal cancer^26,27^, taste impairments^28^ and neurodegenerative diseases^29^.

It has been reported that, in the respiratory tract, TAS2R38 acts as a sentinel in the innate defense by detecting quorum-sensing molecules from pathogens. Its activation stimulates the release of nitric oxide (NO), which has biocidal activity^18,30^. NO and its derivatives, by reducing the palmitoylation of the SARS-CoV-2 nascent spike of protein (S), which affects the fusion with the cognate receptor (angiotensin-converting enzyme 2, ACE-2), reduce the production of viral RNA during the early stages of replication, leading to a less severe syndrome in COVID-19 patients^31,32^. It is interesting to note that in situ hybridization and antibody-specific experiments have shown the presence of ACE2 on type II taste cells, which therefore could be the potential portal for virus entry and this may explain the taste impairments of COVID-19 patients and the vulnerabilities to SARS-CoV-2 in the oral cavity^33^.

The relationship between the severity and duration of COVID-19 symptoms and the TAS2R38 phenotype has been examined in two retrospective investigations, that have suggested an enhanced innate immune protection against SARS-CoV-2 for PROP tasters and super-tasters compared to non-tasters^13,34^. Furthermore, the evaluation of treatment regimens for COVID-19 patients in light of their TAS2R38 phenotype has been proposed to provide additional value for ensuring treatment success^35^. Although a higher presence of the *TAS2R38* PAV haplotype, compared to AVI, has been associated with lower COVID-19 mortality in different countries^36^, another study that assessed the frequency of *TAS2R38* haplotypes in COVID-19 patients with different severity of the disease and in negative subjects, could not confirm the hypothesis that the PAV allele may act as a protecting factor against SARS-CoV-2 infection^37^.

Modifications in sensitivity or gene expression of T2Rs can affect various physiological processes and human pathologies^6^. Environmental exposure can also affect gene expression regulation through DNA methylation (DNAm)^38,39^. Typically, CpG sites are generally unmethylated in the promoters of actively transcribed genes^40^. Moreover, DNAm changes have been associated with various functions and pathologies^41–46^. They also represent a useful indicator of biological aging and early disease risk evaluations for life expectancy and death^47^. The DNAm pattern of genes coding taste receptors and its role in regulating expression and therefore function have not been deeply investigated. Modifications of DNAm of *CD36* and *GPR120* genes have been associated with the detection threshold for fat and bitter taste^48^. DNAm of the *TAS1R2* receptor was linked to carbohydrate intakes and total calories^49^.

TAS2R38 is one of the receptors most implicated in the innate immune response of the respiratory system, and its involvement in COVID-19 infection, severity, and prognosis has not been extensively investigated, and have released inconsistent results. Likewise, to the best of our knowledge, the methylation pattern of *TAS2R38* in nasopharyngeal and/or saliva samples of COVID-19 patients has not been analyzed.

The present study aimed to verify whether changes in *TAS2R38* DNAm patterns can contribute to shedding light on the involvement of TAS2R38 in COVID-19 infection and symptoms severity. To achieve this goal, we analyzed the effect of SARS-CoV-2 on the methylation status of CpG sites associated with *TAS2R38* during infection and after the cessation of the exposition to the virus, also considering the disease severity and the *TAS2R38* genotype of participants. At first, we analyzed *TAS2R38* DNAm profiling in a group of 44 COVID-19-positive patients and 7 participants after the infection (post-COVID-19), who were also included to evaluate the DNAm reversibility after the cessation of the exposition to infection. The *TAS2R38* DNAm profiling was also carried out in a second group of 33 post-COVID-19 participants to analyze the effect of SARS-CoV after the cessation of the exposition to the virus.

**Fig. 1:**
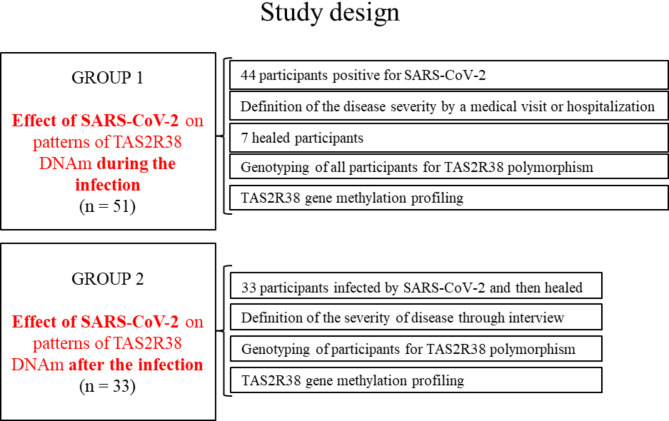
Graphic diagram representing the study design.

The overall design of the study is illustrated in Fig. 1.

## Materials and methods

### Participants

Eighty-four Caucasian subjects were recruited in Sardinia Island, Italy. They consisted of two groups closely similar in age and sex.

The first group included fifty-one participants of which 44 were infected by SARS-CoV-2, as proven by a positive real-time reverse transcriptase-PCR assay (RT-PCR) of a nasopharyngeal swab specimen for SARS-CoV-2 RNA. They displayed severe COVID-19 (*n* = 16) and mild-to-moderate COVID-19 (*n* = 24) or were asymptomatic (*n* = 4). This group included also 7 participants who were post-COVID at the time of sample collection. They were referred to the study from the Santissima Trinità Hospital (Cagliari, Italy) and University Hospital “Policlinico Duilio Casula” (Monserrato, Italy).

The second group included 33 post-COVID-19 participants, as proven by an initial positive real-time reverse transcriptase-PCR assay (RT-PCR) of a nasopharyngeal swab specimen for SARS-CoV-2 RNA, followed by a negative one. They were asked to answer a questionnaire with more detailed questions on their symptoms during the disease, to include them in the following three groups: severe COVID-19 (*n* = 6), mild-to-moderate COVID-19 (*n* = 13), and asymptomatic participants (*n* = 14). They were referred to the study from the Department of Medicine, Surgery and Pharmacy of the University of Sassari, Italy. All participants were categorized based on disease symptoms according to the already validated classification^50^.

All participants were informed about the study’s objectives and methodology and gave their written informed consent. The present work was conducted following the most recent edition of the Helsinki Declaration. For the first group, the protocol was approved by the Ethics Committee of “ATS Sardegna” (224/2020/CE) for the second group, by the University Hospital Company’s (AOU) Ethical Committee in Cagliari, Italy (PG/2021/5471).

### Genotyping for TAS2R38 polymorphisms

For the first group, nasopharyngeal swabs, containing sinonasal epithelial cells that express TAS2R38^18,51^, were collected from participants and immediately stored in a tube with TRIzol reagent (Thermo Fisher Scientific, Waltham, Massachusetts, USA). The protocol of DNA isolation from nasopharyngeal samples is described in^52^. For the second group, DNA was extracted from saliva samples of participants by using the QIAamp^®^ DNA Mini Kit (QIAGEN S.r.l., Milan, Italy) according to the manufacturer’s instructions. Briefly, saliva samples were lysed under denaturing conditions at 56 °C for two hours with proteinase K and a lysis buffer. The lysate was then applied to a silica membrane column, where DNA was selectively bound under denaturing salt conditions. After sequential washes to eliminate impurities and inhibitors, DNA was eluted using 20 µL of elution buffer. The purity and the concentration of purified DNA were estimated by measuring the optical density at 260 nm with a NanoDrop One/One Spectrophotometer (Thermo Fisher Scientific) and by fluorometric reading (Qubit dsDNA BR (Broad-Range) Assay kit). Participants were genotyped for the three single nucleotide polymorphisms (SNPs) *rs713598*,* rs1726866*,* rs10246939* of the *TAS2R38* locus, which cause three amino acid substitutions (Pro49Ala, Ala262Val, and Val296Ile). These three loci result in two major haplotypes, PAV and AVI, and three uncommon haplotypes (AAI, AAV, and PVI). Molecular analyses were carried out by using a TaqMan^®^ SNP Genotyping Assay (C_8876467_10 assay for the *rs713598*; C_9506827_10 assay for the *rs1726866* and C_9506826_10 assay for the *rs10246939*) according to the manufacturer’s specifications (Applied Biosystems by Life Technologies Milano Italia, Europe BV). The reactions were run on 96-well plates with fast thermal cycling conditions and the reagent concentrations were 1X TaqMan^®^ genotyping master mix (code: 4371355), 1X TaqMan^®^ genotyping assays, 10 ng of DNA, and nuclease-free water. The plates were read on a StepOne™ Real-Time PCR System and the results were analyzed by allelic discrimination of the sequence detector software (Genotyping—Applied Biosystems, version v2.3; by Life-Technologies Italia, Europe BV, Monza, Italy). Replicates and positive and negative controls were included in all reactions.

### TAS2R38 gene methylation profiling

Notably, *TAS2R38* is a mono-exonic gene, lacking CpG islands in the promoter region and along the gene body. However, probes interrogating the methylation status of specific CpG sites associated with this gene are included in the commercially available methylation arrays, such as Illumina EPIC arrays. Accordingly, to choose the site where analyze the *TAS2R38* gene methylation profiling, we checked the methylation levels of the CpG loci interrogated by the EPIC arrays in a sub-group of COVID-19 patients previously characterized in a whole-genome methylation study by our research group^52^. One of the CpG locus (cg25481253, chr7:141973584–141973585, hg38), located in the coding region for TAS2R38 receptor, showed a differential methylation level between asymptomatic and symptomatic patients, even more pronounced in those with the most severe symptoms (pneumonia, intubated with high flow oxygen) (Table Sl).

The DNA extracted as described above was bisulfite converted using EZ DNA Methylation Gold Kit ™ (Zymo Research, Irvine, CA, USA) according to the manufacturer’s instructions to bisulfite converts 1000 ng of each DNA sample. Briefly, DNA was incubated with the CT conversion reagent for 10 min at 98 °C followed by a 2.5 h incubation at 64 °C and a final storage step at 4 °C. After the bisulfite clean-up process each sample was collected in a single 1.5 mL Eppendorf tube by flushing the spin column using 10 µL of elution buffer, yielding a final concentration of 100 ng/µL for each sample. Bisulfite-converted DNA was diluted to a final concentration of 10ng/µL, aliquoted, and stored at -20 °C until further processing. The converted DNA was used to determine the *TAS2R38* gene methylation profiling status at the cg25481253 CpG locus.

To detect *TAS2R38* gene methylation, we designed a MethyLight assay which uses primers that hybridize to regions not influenced by methylation (not containing CpG loci) and a probe that hybridizes in the stretch of sequence containing the methylated form CpG site cg25481253. Specifically, a nested PCR approach was employed. The first standard PCR was performed in a conventional thermal cycler and the second one with internal primers was a quantitative MethyLight assay carried out using the primers and probe reported in Table 1. The first-PCR mix solution was prepared to a final volume of 30 µL, containing 30 ng bisulfite-converted DNA, 1X PCR buffer, 50 mM MgCl_2_, 10 mM of total dNTPs, 10 pmol of each primer, and 1 U Platinum™ *Taq* DNA Polymerase, high fidelity (Invitrogen™ Life Technologies, Carlsbad, CA, USA). The amplification reaction was performed with a touchdown method on Rotor-Gene Q (Qiagen, Venlo, Netherlands), with a denaturation step at 94 °C for 2 min and then the denaturation/annealing/extension cycles with annealing temperature decreasing 0.5 °C every cycle from 62 °C to 55 °C, for 12 cycles and then 20 cycles of 94 °C for 30 s, 55 °C for 30 s and 72 °C for 1 min. The initial PCR products were diluted 1000 times with ddH_2_O, and 5 µL of this dilution was used as the DNA template for the qPCR.

**Table 1:** *TAS2R38* methylation assay.

Type	Seq (5′→3′)	Tm (°C)
External_Primer Forward	TTTAATTTTTGGAAGTGGGTAAGTT	58
External_Primer Reverse	AATTTCCTACCAAAACTTTTTATAC	56
Internal_Primer Forward	ATTGTTGTTTAGTGTTTGTTTTTTT	54
Internal_Primer Reverse	ATTCATTTCAATCCTAAAATTTACA	54
Probe	6-FAM TATTAAGAAAACGAAGGTA	63.0

We carried out two qPCR reactions for each sample: one for the target assay and one for the bisulfite-dependent methylation-independent control (ALU-C4) used to normalize the quantity of the input DNA sample^53^. Each reaction was carried out in triplicate and included: 5 µL nested PCR product or 30 ng bisulfite-converted DNA, 1X TaqMan^®^ Genotyping Master mix (Applied Biosystems, Foster City, CA, USA), 900 nM of each primer, and 250 nM of the probe in a final volume of 30 µL. The following temperature settings were used during the experiment on a Rotor-Gene Q (Qiagen, Venlo, Netherlands): 10 min at 95 °C, then 45 cycles of 95 °C for 15 s and 60 °C for 1 min. The methylation levels were expressed as Δ cycle threshold (Ct), which was determined as the difference between Ct of the target assay and Ct of the ALU-C4 control (a lower methylation level is indicated by a higher ΔCt and vice versa).

### Statistical analyses

The genotype distribution and haplotype frequencies of the *TAS2R38* SNPs were tested in the two groups and according to the severity of the disease by the Fisher method (Genepop software version 4.2; online software: http://genepop.curtin.edu.au/genepop_op3.html; Montpellier, France).

Normality testing was done using the Kolmogorov-Smirnov tests. Since the data didn’t respect the normality test, the Kruskal-Wallis test was used to compare ∆Ct values according to *TAS2R38* genotypes and according to the severity of the disease. Post-hoc comparisons were conducted with Dunn’s multiple comparisons test.

Statistical analyses were conducted using STATISTICA for WINDOWS (version 7; StatSoft Inc., Tulsa, OK, USA) and with GraphPad Prism 8 software (GraphPad Software, San Diego, CA, USA). * indicates *P* < 0.05, ** *P* < 0.01 and *** *P* < 0.001 according to the asterisk rating system.

## Results

### *TAS2R38* polymorphism and condition concerning COVID-19

Table 2 shows the distribution of participants according to their *TAS2R38* genotype and condition concerning COVID-19. Nine participants in the first group and one in the second had rare haplotypes and were eliminated from subsequent analyses. The two groups did not differ statistically based on their genotype distribution (χ^2^ = 0.359; *P* = 0.835; Fisher’s test) and haplotype frequencies (χ^2^ = 0.025; *P* = 0.874; Fisher’s test), also considering the condition of participants concerning COVID-19 (χ2 < 3.76; *P* > 0.12; Fisher’s test). No differences related to the severity of the disease were found based on their genotype distribution (first group sample: χ^2^ = 0.910; *P* = 0.634; second group: χ^2^ = 1.772; *P* = 0.412; Fisher’s test) and haplotype frequencies (first group: χ^2^ = 0.879; *P* = 0.644; second group: χ^2^ = 1.946; *P* = 0.378; Fisher’s test).

**Table 2:** Number of participants of group 1 (at the time of infection) and group 2 (after the infection) according to *TAS2R38* genotype and condition concerning COVID-19.

TAS2R38 genotype	Condition of participants concerning COVID-19				
	Total *n* (%)	Severe *n* (%)	Mild-to-moderate *n* (%)	Asymptomatic *n* (%)	Post-COVID *n* (%)
Group 1					
PAV/PAV	8 (19.05)	0 -	6 (28.57)	1 (25.0)	1 (20.0)
PAV/AVI	22 (52.38)	9 (75.0)	9 (42.86)	2 (50.0)	2 (40.0)
AVI/AVI	12 (28.57)	3 (25.0)	6 (28.57)	1 (25.0)	2 (40.0)
Total	42	12	21	4	5
Group 2					
PAV/PAV	7 (21.87)	2 (33.33)	1 (8.33)	4 (28.57)	
PAV/AVI	14 (43.75)	3 (50.0)	5 (41.67)	6 (42.86)	
AVI/AVI	11 (34.38)	1 (16.67)	6 (50.0)	4 (28.57)	
Total	32	6	12	14	

### Effect of SARS-CoV-2 on patterns of* TAS2R38* DNAm during the infection

**Fig. 2:**
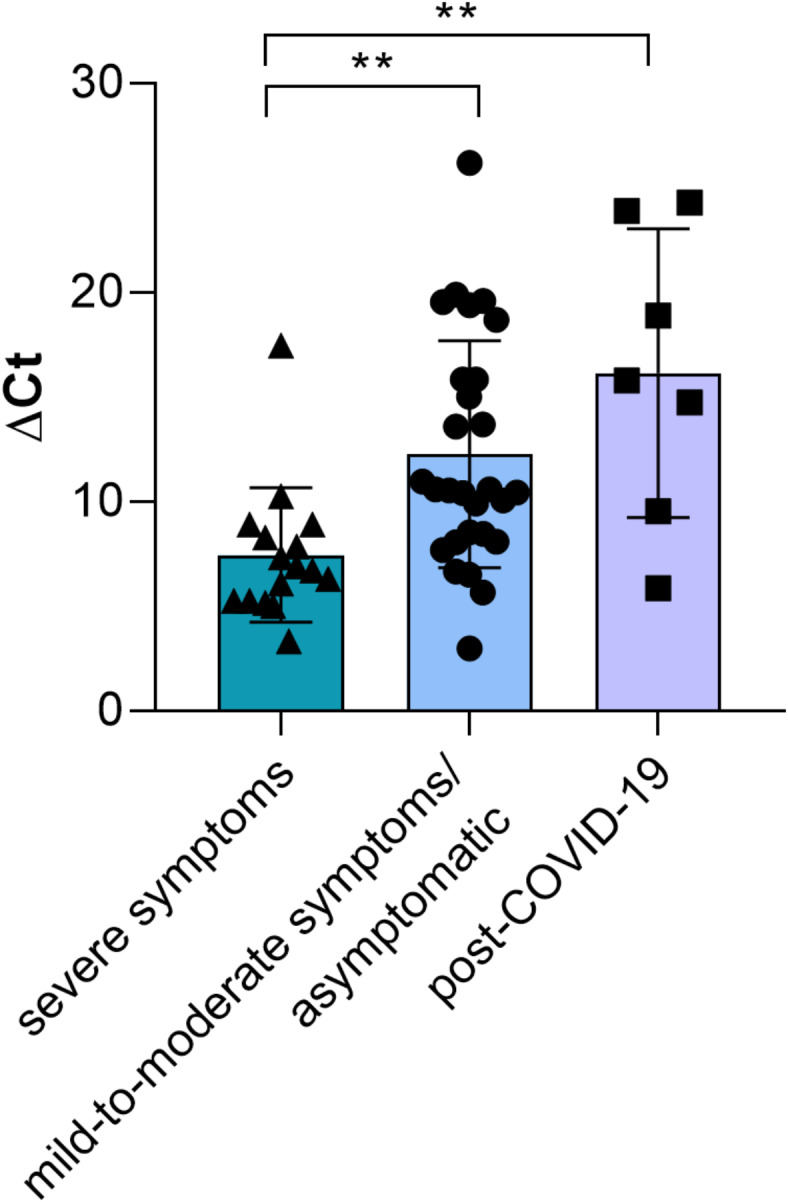
*TAS2R38* DNAm in COVID-19 positive patients with different severity of the disease and post-COVID-19 participants. *n* = 51. Distribution of the ∆Ct values and mean values ± SEM for the COVID-19 patients with severe COVID-19 (*n* = 16), mild-to-moderate COVID-19/asymptomatic (*n* = 28), and post-COVID-19 (*n* = 7) are reported. ** indicates a statistically significant difference (*P* ≤ 0.0031, Dunn’s multiple comparisons test after the Kruskal-Wallis test).

The analysis of the selected CpG locus of COVID-19 positive patients of the first group (*n* = 44), classified into severe (*n* = 16) and asymptomatic/mild-to-moderate (*n* = 28), and 7 post-COVID-19 patients is shown in Fig. 2. The Kruskal-Wallis test showed that the ∆Ct mean values were associated with the condition of participants concerning the disease (H_[2,51]_ = 14.42, *P* = 0.0007, Kruskal-Wallis test). We detected a statistically significant higher methylation level (∆Ct lower) in the severe group compared to other subgroups (*P* = 0.0031 vs. asymptomatic/mild-to-moderate and *P* = 0.0047 vs. post-COVID-19, Dunn’s multiple comparison test). Of note, there is a tendency for a reduction in the mean methylation value in post-COVID-19 patients compared to the asymptomatic/mild-to-moderate subgroup (*P* = 0.99, Dunn’s multiple comparison test).

### Effect of SARS-CoV-2 on patterns of *TAS2R38* DNAm after the infection.

**Fig. 3:**
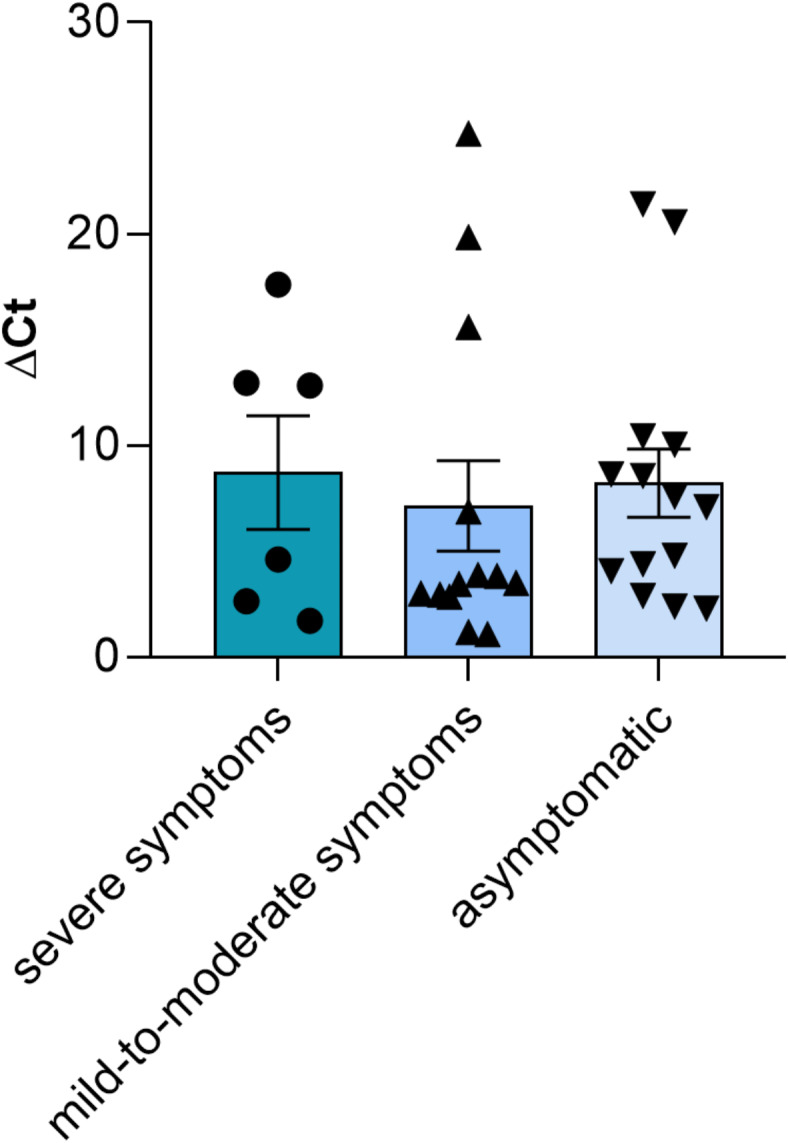
*TAS2R38* DNAm in post-COVID-19 participants, who had previously contracted COVID-19 with different severity of the disease and then recovered. Distribution of the ∆Ct values, and mean values ± SEM, for the participants who had had severe COVID-19 (*n* = 6), mild-to-moderate COVID-19 (*n* = 13), and had been asymptomatic (*n* = 14) are reported. *n* = 33.

The distribution of the ∆Ct values, and mean values ± SEM, determined in the post-COVID-19 participants of the second group, who had contracted COVID-19 with severe symptoms, mild-to-moderate symptoms, and asymptomatic participants is shown in Fig. 3. As expected according to the previous results, we did not observe any statistically significant difference (H_[2,33]_ = 1.423, *P* = 0.4909, Kruskal-Wallis test) among the samples divided according to the symptoms experienced during the time of infection. However, each sample group (severe symptoms, mild-to-moderate symptoms, and asymptomatic) is distributed in two subgroups with different ∆Ct values.

### *TAS2R38* methylation analysis according to the* TAS2R38* polymorphisms

**Fig. 4:**
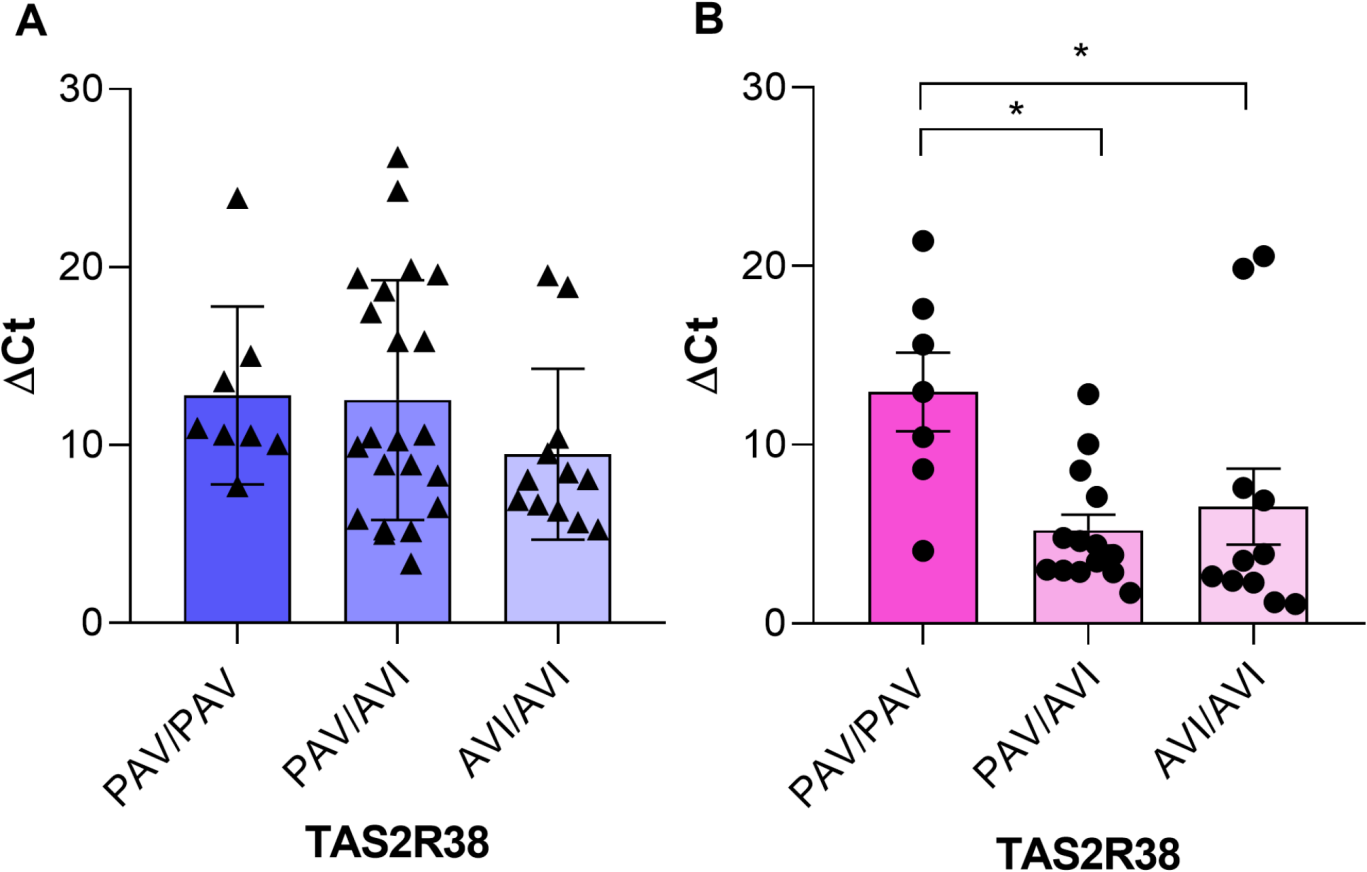
TAS2R38 DNAm related to TAS2R38 genotype. (**A**) Distribution of the ∆Ct values, and mean values ± SEM, for the PAV/PAV (*n* = 8), PAV/AVI (*n* = 22), and AVI/AVI (*n* = 12) participants of the first group at the time of the infection (*n* = 42); (**B**) Distribution of the ∆Ct values, and mean values ± SEM, for the PAV/PAV (*n* = 7), PAV/AVI (*n* = 14), and AVI/AVI (*n* = 11) recovered participants of the second group (*n* = 32). * Indicate a significant difference (*P* ≤ 0.034, Dunn’s multiple comparison test after the Kruskal-Wallis test).

Figure 4 shows the relationship between *TAS2R38* DNAm and *TAS2R38* SNPs genotype of participants of the first group, during infection, and those of the second group, after the cessation of the exposition to the virus. The methylation analysis showed that there was no statistically significant difference in the first group of samples divided according to the SNP genotype i.e. those taking a snapshot of the methylation status at the time of the infection (H_[2,42]_ = 3.578, *P* = 0.1671, Kruskal-Wallis test) (Fig. 4A). On the other hand, *TAS2R38* DNAm in post-COVID-19 participants of the second group, i.e. at the time when there was not any active disease, the ∆Ct mean values were associated with the *TAS2R38* genotype of participants (H_[2,32]_ = 8.514, *P* = 0.0142, Kruskal-Wallis test) (Fig. 4B). The ∆Ct values were significantly higher for those who had PAV/PAV genotype than in heterozygous (*P* = 0.034, Dunn’s multiple comparison test), and AVI homozygous (*P* = 0.019, Dunn’s multiple comparison test).

## Discussion

TAS2R38 is the taste receptor most implicated in the innate immune response of the respiratory system^18,19^. The polymorphisms in the *TAS2R38* gene which affect taste sensitivity also alter the immune responses to upper respiratory infections^18^. Homozygous patients for the PAV variant are less susceptible to respiratory infections than homozygous patients for the AVI variant who have altered TAS2R38-dependent responses, and heterozygous patients. By detecting the bacterial quorum-sensing molecules, the TAS2R38 receptor activates an efficient immune response mediated by NO, which is shown to inhibit the replication of SARS-CoV^54^. Nevertheless, few studies have been conducted on the associations between TAS2R38 and COVID-19 severity and prognosis, and with inconsistent outcomes^13,34,36,37^.

Given that the efficient performance of a receptor depends on its expression which is strongly associated with DNAm^38,39^, the main goal of the current study was to determine whether variations in *TAS2R38* DNAm patterns may shed light on the role of the TAS2R38 bitter taste receptor in COVID-19 infection and the severity of symptoms.

Our results show for the first time, to our knowledge, that cg25481253 methylation level, a CpG site located in the coding region of *TAS2R38*, is positively associated with COVID-19 severity during infection. Interestingly, the methylation pattern returns to a normal state after the infection, as evident from the data obtained from the post-COVID-19 participants in both groups, regardless of the biological matrix used. This is in line with the reversibility of DNA methylation after the cessation of exposure to an environmental trigger^55,56^ and could explain the restoration of taste shown in the post-COVID-19 condition, after a period of ageusia during the infection^57^. Unfortunately, it was impossible to verify this hypothesis in our study. We were unable to collect taste function data in a sufficient number of patients. It would have been interesting to include an outward measure of receptor function to verify whether methylation changes affect the phenotypic expression of the receptor. Therefore, our results, suggesting a role of methylation changes at cg25481253 in regulating TAS2R38 expression, indicate a potential silencing effect of SARS-CoV-2 on TAS2R38 receptor expression that depends on disease severity. The levels of the *TAS2R38* DNAm were significantly higher in positive participants with severe symptoms compared to participants with mild-to-moderate symptoms or those who were asymptomatic and to those who recovered from the disease. From these results, it can be speculated that SARS-CoV-2 like other Coronaviruses^52,58^ can elude the host’s innate immune responses by altering the DNA methylation pattern of genes involved in recognition and response against the pathogen. Interestingly, our results also showed that the methylation pattern returns to a normal state after recovery from the infection, regardless of the severity of the disease, suggesting that the silencing effect depending on severity disappears and the expression of receptor returns to normal, restoring the patient’s immune capacity.

Finally, we found that the levels of the *TAS2R38* DNAm were associated with the *TAS2R38* genotype of participants who were previously infected by SARS-CoV-2 and then recovered. Specifically, participants who had the PAV/PAV genotype showed lower DNAm levels, and therefore a potentially higher expression of the TAS2R38 receptor, compared to heterozygous and AVI homozygous. This result could be interpreted as linkage disequilibrium between the methylation profile and the SNP genotype, observable only when the methylation pattern is not altered by external factors such as viral infections. Unfortunately, we could not compare the ΔCt values of the post-COVID participants in relation to the *TAS2R38* genotype across the two groups because they resulted from a different biological matrix and only five were the post-COVID participants in the first group. It also seems to suggest that the receptor in the PAV form, is a genetic protective condition, not only because it has a higher affinity for the agonist, but also because it may be expressed more. Accordingly, one might speculate that, in case of infection, the PAV variant could activate a prompt and massive immune response which, mediated by NO, could inhibit the replication of SARS-CoV. This could explain why very few PAV/PAV participants showed serious symptoms, none in the first group and only two in the second. On the other hand, the TAS2R38 in the AVI form, would be at a genetic disadvantage, not only because it shows little or no affinity for the agonist, but also because it would be less expressed, further inhibiting its ability to activate the immune response.

In conclusion, our findings unequivocally demonstrate that *TAS2R38* DNAm profiling is crucial for understanding the role of the taste TAS2R38 receptor in COVID-19 severity and might suggest a role of the methylation changes at cg25481253 in the regulation of the TAS2R38 expression. Therefore, the cross-sectional study design allowed us to show that cg25481253 methylation levels were positively associated with COVID-19 severity during infection, but not after the cessation of exposure to an environmental trigger. In addition, although the sample size is small and the statistical significance of the findings is limited, the study provides a valuable reference for understanding the epigenetic mechanisms related to COVID-19 symptoms. This work highlights the need for follow-up studies with larger sample sizes and pre- versus post-infection measurements in the same individuals to further investigate these associations.

## Electronic supplementary material

Below is the link to the electronic supplementary material.

Supplementary Material 1

## Data Availability

All data are available in the main text or the supplementary material or from the corresponding author on reasonable request.
