# Supplementary material for: TAS2R38 gene methylation is associated with syndrome Coronavirus 2 (SARS-CoV-2) infection and clinical symptoms
Source: Sci Rep. 2025 Apr 25;15:14462. doi: 10.1038/s41598-025-95879-x (PMC12032294; doi:10.1038/s41598-025-95879-x)
Supplement: Supplementary file 1 — Supplementary Material 1 [file 41598_2025_95879_MOESM1_ESM.pdf]

Supplementary Materials for

TAS2R38 gene methylation is associated with Syndrome Coronavirus 2 (SARS-CoV-2) infection and clinical symptoms.

Melania Melis[1,*], Eleonora Loi[2], Giacomo Aru[2], Giorgia Sollai[1], Mariano Mastinu[3], Lala Chaimae Naciri[1], Giacomo De Riu[4], Luigi Angelo Vaira[4], Giulia Costanzo[5], Davide Firinu[5], Paola Cabras[6], Aldo Caddori[6], Roberto Crnjar[1], Patrizia Zavattari[2] and Iole Tomassini Barbarossa[1]

[1]Department of Biomedical Sciences, Unit of Physiology, University of Cagliari, 09042 Monserrato (CA), Italy.
[2]Department of Biomedical Sciences, Unit of Biology and Genetics, University of Cagliari, 09042 Cagliari, Italy.
[3]Smell & Taste Clinic, Department of Otorhinolaryngology, Technical University of Dresden, 01307 Dresden, Germany.
[4]Maxillofacial Surgery Unit, Department of Medicine, Surgery and Pharmacy, University of Sassari, 07100 Sassari, Italy.
[5]Department of Medical Sciences and Public Health, Monserrato Campus, University of Cagliari, 09042 Cagliari, Italy.
[6]Department of Internal Medicine, Hospital SS. Trinità, ATS, Cagliari, Italy.

*Corresponding author. Email: melaniamelis@unica.it

Preliminary result from the whole genome methylation study of nasopharyngeal samples

The methylation levels of 15 CpG loci associated with *TAS2R38* were checked in a small group of nasopharyngeal samples subjected to a genome-wide methylation assay[1] finding one altered CpG site in different comparisons (Table S1). We detected a tendency to hypermethylation in COVID-19 patients compared to controls, subjects who were negative for SARS-CoV-2 at the time of sample collection. Considering symptomatology, we found that symptomatic patients were hypermethylated respect to the asymptomatic even more pronounced in patients with a severe condition. Of note, controls, asymptomatic and patients with mid-to-moderate symptoms showed the same methylation level.

Table S1. cg25481253 methylation level and differential methylation values in the COVID-19 patients, classified according to the disease severity and controls previously analyzed in Loi et al 2022 [1]

<table>
<tr><th></th><th>cg25481253 methylation value</th><th>Differential methylation value</th></tr>
<tr><td>COVID-19 (n=13)</td><td>0.63</td><td rowspan="2">0.06</td></tr>
<tr><td>Controls (n=2)</td><td>0.57</td></tr>
<tr><td></td><td></td><td></td></tr>
<tr><td>Symptomatic (n=8)</td><td>0.68</td><td rowspan="2">0.11</td></tr>
<tr><td>Asymptomatic (n=5)</td><td>0.57</td></tr>
<tr><td></td><td></td><td></td></tr>
<tr><td>Severe (n=4)</td><td>0.78</td><td rowspan="2">0.21</td></tr>
<tr><td>Mild-to-moderate (n=4)</td><td>0.57</td></tr>
</table>

All comparisons were not significant in the differential methylation analysis performed using RnBeads[2,3] ($P > 0.05$).

References

1. Loi, E. *et al.* HLA-C dysregulation as a possible mechanism of immune evasion in SARS-CoV-2 and other RNA-virus infections. *Front Immunol* **13**, 1011829, doi:10.3389/fimmu.2022.1011829 (2022).
2. Assenov Y, Müller F, Lutsik P, Walter J, Lengauer T, Bock C. Comprehensive analysis of DNA methylation data with RnBeads. *Nat Methods* **11**, 1138–40. doi: 10.1038/nmeth.3115 (2014).
3. Müller F, Scherer M, Assenov Y, Lutsik P, Walter J, Lengauer T, et al. RnBeads 2.0: Comprehensive analysis of DNA methylation data. *Genome Biol* **20**, 55. doi: 10.1186/s13059-019-1664-9 (2019).
